# Comparison of retinal vascular geometry in obese and non-obese children

**DOI:** 10.1371/journal.pone.0191434

**Published:** 2018-02-01

**Authors:** Evelyn Li Min Tai, Yee Cheng Kueh, Wan-Hazabbah Wan Hitam, Tien Yin Wong, Ismail Shatriah

**Affiliations:** 1 Department of Ophthalmology, School of Medical Sciences, Health Campus, Universiti Sains Malaysia, Kubang Kerian, Kelantan, Malaysia; 2 Hospital Universiti Sains Malaysia, Kubang Kerian, Kelantan, Malaysia; 3 Unit of Biostatistics & Research Methodology, School of Medical Sciences, Health Campus, Universiti Sains Malaysia, Kubang Kerian, Kelantan, Malaysia; 4 Singapore Eye Research Institute, Singapore National Eye Centre, Singapore, Singapore; 5 DUKE-NUS Medical School, Singapore, Singapore; 6 Centre for Eye Research Australia, University of Melbourne, Melbourne, Australia; USF Health Morsani College of Medicine, UNITED STATES

## Abstract

**Purpose:**

Childhood obesity is associated with adult cardiometabolic disease. We postulate that the underlying microvascular dysfunction begins in childhood. We thus aimed to compare retinal vascular parameters between obese and non-obese children.

**Methods:**

This was a cross-sectional study involving 166 children aged 6 to 12 years old in Malaysia. Ocular examination, biometry, retinal photography, blood pressure and body mass index measurement were performed. Participants were divided into two groups; obese and non-obese. Retinal vascular parameters were measured using validated software.

**Results:**

Mean age was 9.58 years. Approximately 51.2% were obese. Obese children had significantly narrower retinal arteriolar caliber (F_(1,159)_ = 6.862, p = 0.010), lower arteriovenous ratio (F_(1,159)_ = 17.412, p < 0.001), higher venular fractal dimension (F_(1,159)_ = 4.313, p = 0.039) and higher venular curvature tortuosity (F_(1,158)_ = 5.166, p = 0.024) than non-obese children, after adjustment for age, gender, blood pressure and axial length.

**Conclusions:**

Obese children have abnormal retinal vascular geometry. These findings suggest that childhood obesity is characterized by early microvascular abnormalities that precede development of overt disease. Further research is warranted to determine if these parameters represent viable biomarkers for risk stratification in obesity.

## Introduction

The prevalence of childhood obesity has been increasing worldwide [1, 2]. Childhood obesity has adverse long-term health implications, especially for cardiometabolic conditions like hypertension, diabetes and ischaemic heart disease [3]. The underlying pathology in these diseases occurs at the level of the microvasculature [4, 5].

Obesity-related endothelial function has been observed in both animal and human studies. Among obese rats, obesity has been found to increase endothelial susceptibility to hyperglycaemia-induced oxidative stress [6]. Interestingly, animal models have shown that this protein kinase C-mediated endothelial damage is also central to the pathogenesis of diabetic microvascular complications [7] such as retinopathy [8, 9] and nephropathy [10]. This pathway is likewise operative in humans, in whom protein kinase C blockade reduces endothelial dysfunction secondary to hyperglycaemia, both in diabetic [11] and non-diabetic subjects [12].

Although obesity is often linked with metabolic disease, increased body weight per se is independently associated with impaired endothelium-related coronary vessel function [13]. Healthy obese adults have been noted to have poorer endothelial function than their non-obese counterparts [14–16]. Similarly, endothelial dysfunction has been demonstrated in obese children [17, 18]. In these children, compensatory elevation of circulating endothelial progenitor cell counts suggest that in early life, obesity-mediated endothelial dysfunction may still be reversible [19].

Microvasculature abnormalities in obesity may be visualized non-invasively via digital retinal vessel analysis [20]. Based on the optimum design principle, the architecture of the human microvasculature is designed to be energy-efficient, i.e, to provide adequate perfusion with the minimum of energy expenditure [21]. Retinal vascular geometry not only reflects the complexity of the vascular tree, but also provides insight into the ‘optimality’ of the microcirculation [22]. Retinal vascular caliber has previously been shown to be abnormal in obese adults [23]. These changes begin even in childhood, with an inverse association between body mass index and arteriolar caliber [24, 25]. Newer retinal vascular parameters, such as fractal dimension, branching coefficient, and tortuosity have been associated with obesity-linked microvascular diseases including hypertension [26], diabetes [27] and stroke [28]. However, few studies have examined the effect of obesity per se on these novel vessel parameters [24, 29]. Our study thus aimed to compare retinal vascular geometry between obese and non-obese children.

## Materials and methods

### Study population

This was a cross-sectional prospective study conducted in the Eye Clinic of Hospital Universiti Sains Malaysia between January 2015 and March 2016. A total of 166 children aged 6 to 12 years old were recruited. The study was approved by the Human Research Ethics Committee of Universiti Sains Malaysia. The conduct of the study followed the tenets of the declaration of Helsinki.

Children who fulfilled the inclusion and exclusion criteria were invited to participate. The inclusion criteria was age between 6 and 12 years old at the time of examination, a best corrected visual acuity better than 6/12, and a normal eye examination. Exclusion criteria was strabismus, amblyopia, optic nerve abnormalities, high refractive errors (based on spherical equivalent of ±4.0 diopters], history of ocular trauma, ocular pathology, developmental delay, and systemic illnesses like diabetes or hypertension. Informed written consent was obtained from at least one parent, as well as verbal assent from the child.

### Ocular examination, refraction and axial length measurement

Upon arrival at the eye clinic, distance visual acuity was assessed monocularly using a Snellen chart for distance (Reichert; NY) at six meters. A comprehensive eye examination including pupillary examination, anterior segment examination and complete retinal evaluation was performed. An autokeratorefractometer (Model RK5; Canon Inc, Tokyo, Japan) was used to obtain three consecutive readings of sphere and cylinder, with a maximum acceptable difference of 0.25 diopters between the lowest and highest readings. Spherical equivalent was calculated as the value of the sphere plus half of the value of the cylinder. The right eye axial length was measured using a non-contact partial coherence interferometer (IOL Master, Carl Zeiss Meditec; Jena, Germany). The mean axial length was derived from a mean of five consecutive readings. An acceptable reading had a signal-to-noise ratio of more than 2 mm, and a difference of 0.05 mm or less between the lowest and highest reading.

### Anthropometric measurements

Blood pressure was measured in the sitting position after 5 minutes of rest, using a digital automated sphygmomanometer (Model SEM-1 [HEM-7051-C12], Omron Healthcare Co., Ltd.; Kyoto, Japan) with an appropriate-sized cuff. The average systolic and diastolic blood pressure was obtained from two readings. A third blood pressure reading would be obtained if the difference between the first two readings were greater than 10 mm Hg in systolic blood pressure (SBP) and/or 5 mm Hg in diastolic blood pressure (DBP).

Height and weight were measured with a height and weight measuring scale (Model 220, Seca; Hamburg, Germany) according to standard protocols. Height was recorded to the nearest 1 mm. Weight was recorded to the nearest 0.1 kg. Body mass index (BMI) was calculated as weight divided by the height squared (kg per meter squared). Obesity was classified as BMI of > 2 SD (standard deviation) above the mean, based on World Health Organization age and sex-specific growth charts.

### Retinal examination and vascular analyses

Pupil dilation was achieved with a single drop of topical phenylephrine 2.5% and tropicamide 1%, after which 45 degree optic disc-centered retinal photographs were taken of both eyes using a digital fundus camera (Model VX-10, Kowa; Tokyo, Japan). If both images were of equivalent quality, the image from the right eye was selected. A single grader, masked to participant identity, performed the retinal vessel analysis using a validated semi-automated computed software, SIVA (Singapore I Vessel Assessment; National University of Singapore, Singapore).

**Fig 1:**
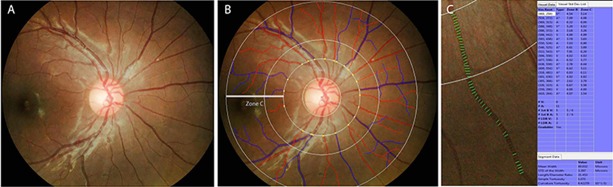
Fundus photo centered on the optic disc in Singapore I Vessel Assessment (SIVA) software. Panel A shows an example of a digital fundus photo pre-processing. Panel B shows a screenshot of the SIVA system. All retinal vessels greater than 25 um in diameter located between one-half to two disc diameters from the optic disc margin (i.e. in Zone C) are outlined and their edges marked using a pixel density histogram. The retinal arterioles are outlined in red, while the venules are outlined in blue. In Panel C, the green lines overlying the segment of a vessel are referred to as ‘covers’. A minimum of 5 covers are needed; based on these, the software will then provide an average of the mean retinal arteriolar and venular calibers.

SIVA is a semi-automated program in which all retinal vessels greater than 25 um in diameter located between one-half to two disc diameters from the optic disc margin are outlined and their edges marked using a pixel density histogram. Retinal vascular parameters are estimated based on measurements of the biggest six arterioles and venules in this area (zone C) (Fig 1). Based on the Knudtson-Parr-Hubbard formula, the average retinal arteriolar and venular caliber were calculated and summarized as the central retinal arteriolar equivalent (CRAE) and central retinal venular equivalent (CRVE) [30]. The software also automatically provided their ratio (arteriovenous ratio, AVR). Correction for the effect of ocular magnification on vessel sizes was performed using the Bengtsson formula [31].

Retinal fractal dimension (Df) is calculated from the outlined retinal vessels using the ‘box-counting method’. In this method, the digital retinal image is compartmentalized into equally-sized squares (i.e. boxes), then the number of squares containing the skeletonized (i.e outlined) segments of retinal vessels is calculated [32]. The process is then repeated with squares of differing sizes. The fractal dimension is the gradient of the logarithm of the number of squares through which the vessel outline passes against the logarithm of the size of the square. Larger values represent a more complex branching pattern.

**Fig 2:**
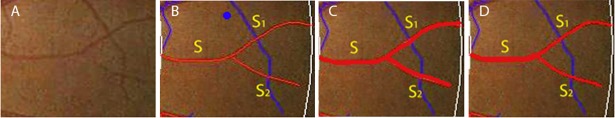
Diagrammatic illustration of branching coefficient. A portion of the retinal arteriole nasal to the disc is shown, pre (panel A) and post-processing (panel B) by SIVA. Branching coefficient (BC) is calculated as $BC=\frac{\left(S_{1}^{2}+S_{2}^{2}\right)}{S^{2}}$, where S is the root, or main segment of vessel, and S_1_ and S_2_ are its branches. Note the relative thicknesses of the main segment of the vessel compared to its branches, as artificially illustrated in panel C and D. A higher BC reflects similarly sized vessel diameters between the main vessel and its branches (panel C), while a lower BC is related to a decrease in the diameters of the branches compared to the main vessel (panel D).

Branching coefficient (BC) is a method of estimating the ratio between the diameters of the main vessel and the diameters of its branches, or ‘daughter vessels’. It is given by the area ratio: $BC=\frac{\left(S_{1}^{2}+S_{2}^{2}\right)}{S^{2}}$, where S is the root, or main segment of vessel, and S_1_ and S_2_ are its branches [33]. A higher BC reflects similarly sized vessel diameters between the main vessel and its branch, while a lower BC is related to a decrease in the diameters of the branches compared to the main vessel (Fig 2).

Retinal tortuosity is an index, represented as simple tortuosity (sTORT) and curvature tortuosity (cTORT). STORT is calculated by the actual length of vessel divided by the Euclidean distance between the first and last points of that vessel (i.e., the length of the straight line connecting the two points) [34]. CTORT is defined as the integral of curvature square along the path of the vessel divided by the total arc length [35]. A lower tortuosity index represents straighter vessels.

### Statistical analyses

Statistical analyses were performed using IBM SPSS Statistics for Windows, Version 24.0 (IBM Corp, Armonk, NY). Chi-square test was used to determine the association between obese, non-obese children and gender. Independent t-test was used to determine the mean differences of numerical variables between obese and non-obese children. Analysis of covariance (ANCOVA) was performed to determine the mean differences in dependent variables (i.e., caliber, Df, AVR, BC, sTORT, cTORT) between obese and non-obese children, with adjustment for possible confounding variables (i.e., age, gender, SBP, DBP and axial length) [36]. Several models were tested for each dependent variable with correction for confounding variables in ANCOVA; Model 1 was adjusted for age and gender, Model 2 was adjusted for age, gender, SBP and DBP, and Model 3 was adjusted for age, gender, SBP, DBP, and axial length. *P* values of <0.05 were considered statistically significant.

## Results

A total of 166 Malay children were included in this study. Their mean age was 9.58 years. Approximately 50% were female. 51.2% were categorized as obese. Other systemic demographics and retinal vascular parameters are summarized in Table 1. Obese children had a significantly lower retinal arteriolar caliber and arteriovenous ratio than non-obese children. Conversely, the venular Df, BC and cTORT were significantly higher in obese children than in non-obese children.

**Table 1:** Characteristics of participants in obese and non-obese groups.

Variables		Obese (n = 85) Mean ± SD	Non-obese (n = 81) Mean ± SD	t-statistic (df = 164)	p-value
Gender:	Boy	47 (55.3%)^a^	33 (40.7%)^a^	3.52 (1)^b^	0.06^b^
	Girl	38 (44.7%)^a^	48 (59.3%)^a^		
Age, years		9.53 ± 1.62	9.64 ± 1.75	-0.43	0.668
SBP, mm Hg		109.40 ± 11.84	107.53 ± 11.55	1.03	0.304
DBP, mm Hg		69.95 ± 10.24	65.27 ± 8.39	3.21	0.002*
Axial length, mm		23.22 ± 0.80	23.01 ± 0.75	1.74	0.084
Retinal Vascular Parameters:					
Caliber	Arterial	165.84 ± 11.87	172.39 ± 13.25	3.36	0.001*
	Venular	253.41 ± 18.66	250.35 ± 16.22	-1.13	0.261
Arteriovenous ratio		0.66±0.04	0.69±0.05	-4.83	<0.001*
Fractal dimension:	Arterial	1.21 ± 0.05	1.22 ± 0.05	-1.44	0.152
	Venular	1.21 ± 0.05	1.19 ± 0.05	2.04	0.043*
Branching coefficient:	Arterial	1.41 ± 0.41	1.43 ± 0.27	-0.37	0.716
	Venular	1.33 ± 0.32	1.24 ± 0.25	2.05	0.042*
Simple tortuosity:	Arterial	1.10 ± 0.02	1.09 ± 0.02	1.46	0.146
	Venular	1.10 ± 0.01	1.10 ± 0.02	0.568	0.571
Curvature tortuosity:	Arterial	6.41 ± 1.04	6.24 ± 1.11	1.06	0.292
	Venular	6.73 ± 0.90	6.43 ± 0.71	2.37	0.019*

SBP, systolic blood pressure; DBP, diastolic blood pressure; SD, standard deviation

^a^Frequency (percentage)

^b^Chi-square test and its p-value.

*Statistical difference (p < 0.05) between the obese and non-obese group

The differences in the arteriolar caliber, AVR, venular Df and venular cTORT between obese and non-obese children remained significant after adjusting for age, gender, SBP, DBP and axial length (Table 2). After multivariable adjustment, there was no significant difference in venular BC between obese and non-obese children.

**Table 2:** Comparison of retinal vascular parameters (caliber, fractal dimension, branching coefficient, tortuosity) between obese and non-obese children.

Retinal Vascular Parameters		Obese (n = 85) Adjusted mean (95% CI)	Non-obese (n = 81) Adjusted mean (95% CI)	Mean difference (95% CI)	F (df)	*P* value
Caliber:						
Arterial	Model 1	166.035 (163.348, 168.721)	172.046 (169.278, 174.813)	-6.011 (-9.889, -2.133)	9.369 (1, 162)	0.003*
	Model 2	166.196 (163.483, 168.909)	171.879 (169.081, 174.677)	-5.683 (-9.669, -1.697)	7.928 (1, 160)	0.005*
	Model 3	166.557 (163.983, 169.132)	171.581 (168.927, 174.234)	-5.023 (-8.810, -1.236)	6.862 (1, 159)	0.010*
Venular	Model 1	253.544 (249.765, 257.322)	250.118 (246.225, 254.011)	3.426 (-2.030, 8.881)	1.538 (1, 162)	0.217
	Model 2	253.521 (249.658, 257.385)	250.144 (246.159, 254.129)	3.377 (-2.299, 9.054)	1.380 (1, 160)	0.242
	Model 3	253.873 (250.090, 257.657)	249.853 (245.954, 253.752)	4.020 (-1.545, 9.585)	2.036 (1, 159)	0.156
Arteriovenous ratio	Model 1	0.656 (0.647, 0.666)	0.689 (0.679, 0.699)	-0.033 (-0.046, -0.019)	21.288 (1, 162)	<0.001*
	Model 2	0.657 (0.647, 0.667)	0.688 (0.678, 0.698)	-0.031 (-0.046, -0.017)	18.392 (1, 160)	<0.001*
	Model 3	0.657 (0.648, 0.667)	0.688 (0.678, 0.698)	-0.030 (-0.045, -0.016)	17.412 (1, 159)	<0.001*
Fractal dimension:						
Arterial	Model 1	1.206 (1.196, 1.217)	1.219 (1.208, 1.229)	-0.012 (-0.027, 0.003)	2.597 (1, 162)	0.109
	Model 2	1.207 (1.196, 1.217)	1.218 (1.207, 1.229)	-0.011 (-0.027, 0.004)	2.052 (1, 160)	0.154
	Model 3	1.207 (1.197, 1.218)	1.218 (1.207, 1.228)	-0.010 (-0.026, 0.005)	1.721 (1, 159)	0.191
Venular						
	Model 1	1.208 (1.198, 1.218)	1.195 (1.184, 1.205)	0.013 (-0.001, 0.028)	3.158 (1, 162)	0.077
	Model 2	1.208 (1.198, 1.218)	1.194 (1.184, 1.205)	0.014 (-0.001, 0.029)	3.454 (1, 160)	0.065
	Model 3	1.209 (1.199, 1.219)	1.193 (1.183, 1.204)	0.015 (0.001, 0.030)	4.313 (1, 159)	0.039*
Branching coefficient:						
Arterial	Model 1	1.409 (1.335, 1.484)	1.433 (1.356, 1.509)	-0.023 (-0.131, 0.084)	0.184 (1, 162)	0.669
	Model 2	1.413 (1.338, 1.489)	1.429 (1.350, 1.507)	-0.015 (-0.127, 0.096)	0.072 (1, 160)	0.789
	Model 3	1.414 (1.338, 1.490)	1.428 (1.349, 1.507)	-0.014 (-0.126, 0.098)	0.061 (1, 159)	0.805
Venular	Model 1	1.323 (1.262, 1.385)	1.247 (1.183, 1.310)	0.076 (-0.012, 0.165)	2.891 (1, 162)	0.091
	Model 2	1.314 (1.252, 1.377)	1.256 (1.192, 1.321)	0.058 (-0.034, 0.150)	1.563 (1, 160)	0.213
	Model 3	1.314 (1.251, 1.376)	1.257 (1.192, 1.321)	0.057 (-0.035, 0.150)	1.496 (1, 159)	0.223
Simple tortuosity:						
Arterial	Model 1	1.099 (1.095, 1.104)	1.094 (1.089, 1.098)	0.006 (0.001, 0.012)	3.352 (1, 162)	0.069
	Model 2	1.099 (1.095, 1.103)	1.094 (1.090, 1.099)	0.005 (-0.002, 0.011)	2.200 (1, 160)	0.140
	Model 3	1.099 (1.095, 1.103)	1.094 (1.090, 1.098)	0.005 (-0.001, 0.011)	2.546 (1, 159)	0.113
Venular	Model 1	1.097 (1.094, 1.100)	1.096 (1.093, 1.099)	0.001 (-0.003, 0.005)	0.144 (1, 162)	0.705
	Model 2	1.097 (1.094, 1.100)	1.096 (1.093, 1.099)	0.001 (-0.003, 0.005)	0.226 (1, 160)	0.635
	Model 3	1.097 (1.095, 1.100)	1.096 (1.093, 1.099)	0.002 (-0.003, 0.006)	0.500 (1, 159)	0.481
Curvature tortuosity:						
Arterial	Model 1	6.437 (6.209, 6.665)	6.200 (5.964, 6.435)	0.237 (-0.092, 0.567)	2.019 (1, 161)	0.157
	Model 2	6.426 (6.194, 6.658)	6.211 (5.971, 6.452)	0.214 (-0.127, 0.556)	1.535 (1, 159)	0.217
	Model 3	6.428 (6.195, 6.662)	6.209 (5.967, 6.451)	0.220 (-0.124, 0.563)	1.593 (1, 158)	0.209
Venular	Model 1	6.727 (6.551, 6.903)	6.436 (6.254, 6.618)	0.291 (0.036, 0.545)	5.092 (1, 161)	0.025*
	Model 2	6.727 (6.549, 6.905)	6.436 (6.251, 6.620)	0.291 (0.030, 0.553)	4.832 (1, 159)	0.029*
	Model 3	6.733 (6.555, 6.911)	6.431 (6.246, 6.615)	0.302 (0.040, 0.565)	5.166 (1, 158)	0.024*

CI, confidence interval; Model-1, adjusted for age and gender; Model-2, adjusted for age, gender, SBP, DBP; Model-3, adjusted for age, gender, SBP, DBP and axial length

*Statistical difference (p < 0.05) between the obese and non-obese group

## Discussion

Childhood obesity is a risk factor for various diseases, notably diabetes [37]. Diabetic patients have previously been observed to have abnormal retinal vascular geometry [38]. However, among obese individuals, data regarding these vessel parameters is scarce. Retinal vessel analysis in obese children may identify early changes prior to the development of obesity-linked microvascular disease. Our study demonstrates unique differences in retinal vascular parameters between obese and non-obese primary school children.

We found that obese children had significantly narrower retinal arterioles than non-obese children. Among studies which have evaluated the effect of BMI on retinal vessels, significant differences in retinal arteriolar caliber have been noted between subjects in the lowest and highest BMI quartiles [24, 25]. Although obese children showed a trend towards wider venules, this difference was not statistically significant, which is consistent with the literature [39, 40]. BMI may have an indirect effect on the microvasculature via its association with increased blood pressure [41, 42], but our finding that the differences in retinal arteriolar caliber between obese and non-obese children persisted after adjustment for blood pressure supports the hypothesis that the arteriolar narrowing in obese children represents impaired vasodilatory function [20]. These results are substantiated in animal studies; Frisbee et al demonstrated that in obese Zucker rats, there is enhancement of vasoconstrictor processes and impairment of endothelium-dependent vasodilator responses [43]. The latter process may be mediated by nitric oxide (NO) [44], as evidenced by studies showing NO-dependent improvement in microvascular function after therapeutic interventions in obese rats [45, 46].

We observed that venular Df was significantly higher in obese children than in non-obese children. These results differ from those of Gopinath et al, who found that fractal dimension was not significantly associated with body mass index [24]. However, the authors later noted that carbohydrate intake and a high-glycaemic index diet were associated with greater retinal Df in girls [47]. As nutrition and body mass index are inextricably intertwined, obese children may have greater Df due to a complex interplay between these factors and the microvasculature. Df is a proxy for the geometric complexity of the retinal branching pattern, and is associated with hypertension [48] and lacunar stroke [28]. Diabetic patients have been observed to have higher Df than controls [49]. We postulate that the higher Df in obese children may represent microvascular alterations preceding the development of diabetes and its associated complications.

Retinal venular BC was higher in obese children than among non-obese children, but after multivariable adjustment, the significance of these associations disappeared. To our knowledge, no previous study has explored the relationship between obesity and BC. Venular BC increases when the area of the branch venules increases disproportionately to that of the main vessel. The selective effect on these venules is attributed to the fact that unlike in arterioles, where wall shear stress is lower in second-order arterioles, shear stress, such as occurs with elevated blood pressure, has been found to be similar in first and second-order venules [50]. The thinner walls of second-order venules may less resistant to stress than first-order venules, resulting in endothelial inflammation. In rat models, inflammation-induced vasodilation predominantly affects venules, substantiating our hypothesis [51]. Endothelial inflammation also disrupts the delicate balance between reactive oxygen species and antioxidant defenses, resulting in damaged endothelial cells [52]. As subclinical endothelial dysfunction is present in obese children, the underlying pathogenesis may be as discussed above [53].

Suboptimal BC is not only an indirect measure of endothelial dysfunction; it is also associated with altered shear stress across the retinal vascular network, thus propagating a vicious cycle of inflammation and further injury [54]. The increased stress on the vasculature may be compounded by various systemic factors, which may explain the lack of statistical significance in BC after adjustment for confounders. Suboptimal BC has been linked to impairment in general cognitive ability and verbal fluency [55]. Although the association of BC with cognition has not been specifically assessed in obese children, obese individuals have been found to have poorer cognitive ability than controls [56]. Further studies are required to determine whether these findings are reflective of the suboptimal BC in obese patients.

Although our STORT values were similar in both groups, we found a significantly higher venular cTORT in obese than non-obese children. These results differed from those of Sasongko et al, in which no association of body mass index with tortuosity was observed [29]. As sTORT cannot distinguish between true tortuosity (multiple points of inflection) and increased length of the vessel due to bowing, cTORT may be a more accurate measure of vessel tortuosity [57]. Increased retinal venular tortuosity is associated with diabetic retinopathy [58] and cognitive impairment [59]. Vessels become more tortuous in response to increased transmural pressure [60], which may explain the association of tortuosity with blood pressure [26]. The selective increase in venular tortuosity may be attributed to the relative paucity of smooth muscle in venular walls, making them more vulnerable to distortion than arterioles, which have a more developed tunica media. Furthermore, retinal arteriolar autoregulation in response to various factors such as pressure, shear stress and metabolic demand may also explain the observed lack of association of arteriolar parameters with obesity [61].

Evaluation of retinal vascular geometry in a cohort of obese children free of other systemic disease enables non-invasive identification of early retinal microvascular alterations prior to the development of overt disease. Our study confirms the previous observations of retinal arteriolar narrowing in obesity, and identifies novel abnormalities in venular Df and cTORT among obese children. The strengths of this study include its objective quantification of retinal vascular geometry via a semi-automated, validated computer program, its sampling of subjects from a single ethnic group, adjustment for multiple confounders and the use of vessel indices that are independent of magnification error and cardiac cycle [62]. However, the cross-sectional nature of this study limits our ability to make inferences of a temporal nature, and body mass index merely acts as a substitute for obesity. Combining body mass index with other adiposity-related measures such as fat mass by skin-fold thickness may strengthen the clinical significance of these findings [40, 63]. There is also a need for prospective, longitudinal studies to demonstrate the sequential changes of the microvasculature which occur in the development of obesity-related disease.

## Conclusion

Obese children have abnormal retinal vascular geometry. These findings suggest that the microvascular abnormalities observed in obesity-related diseases like diabetes have their origins in childhood. Retinal vascular geometry may thus represent a biomarker for risk stratification, as well as a potential therapeutic target for obesity interventions.

## Supporting information

S1 Table Systemic, ocular and retinal vascular parameters of study subjects. (DOCX)

## References

[1] Skinner AC, Perrin EM, Skelton JA. Prevalence of obesity and severe obesity in US children, 1999–2014. Obesity (Silver Spring). 2016;24(5):1116–23. 27112068 10.1002/oby.21497

[2] Zhang YX, Chu ZH, Li SY, Zhao JS, Zhou JY. Trends in the Prevalence of Morbid Obesity among Children and Adolescents in Shandong, China, 1995–2014. J Trop Pediatr. 2017. 10.1093/tropej/fmx030 28419373

[3] Reilly JJ, Kelly J. Long-term impact of overweight and obesity in childhood and adolescence on morbidity and premature mortality in adulthood: systematic review. Int J Obes. 2011;35(7):891–8. 10.1038/ijo.2010.222 20975725

[4] Cheung CY, Ikram MK, Sabanayagam C, Wong TY. Retinal microvasculature as a model to study the manifestations of hypertension. Hypertension. 2012;60(5):1094–103. doi: 10.1161/HYPERTENSIONAHA.111.189142 23045470 10.1161/HYPERTENSIONAHA.111.189142

[5] Cheung CY, Ikram MK, Klein R, Wong TY. The clinical implications of recent studies on the structure and function of the retinal microvasculature in diabetes. Diabetologia. 2015;58(5):871–85. doi: 10.1007/s00125-015-3511-1 25669631 10.1007/s00125-015-3511-1

[6] Bohlen HG, Nase GP. Obesity lowers hyperglycemic threshold for impaired in vivo endothelial nitric oxide function. Am J Physiol Heart Circ Physiol. 2002;283(1):H391–7. doi: 10.1152/ajpheart.00019.2002 12063313 10.1152/ajpheart.00019.2002

[7] Way KJ, Katai N, King GL. Protein kinase C and the development of diabetic vascular complications. Diabet Med. 2001;18(12):945–59. 11903393 10.1046/j.0742-3071.2001.00638.x

[8] Park J, Kim H, Park SY, Lim SW, Kim YS, Lee DH, et al Tonicity-responsive enhancer binding protein regulates the expression of aldose reductase and protein kinase C delta in a mouse model of diabetic retinopathy. Exp Eye Res. 2014;122:13–9. doi: 10.1016/j.exer.2014.03.001 24631337 10.1016/j.exer.2014.03.001

[9] Song HB, Jun HO, Kim JH, Yu YS, Kim KW, Kim JH. Suppression of protein kinase C-zeta attenuates vascular leakage via prevention of tight junction protein decrease in diabetic retinopathy. Biochem Biophys Res Commun. 2014;444(1):63–8. doi: 10.1016/j.bbrc.2014.01.002 24434146 10.1016/j.bbrc.2014.01.002

[10] Menne J, Shushakova N, Bartels J, Kiyan Y, Laudeley R, Haller H, et al Dual inhibition of classical protein kinase C-alpha and protein kinase C-beta isoforms protects against experimental murine diabetic nephropathy. Diabetes. 2013;62(4):1167–74. doi: 10.2337/db12-0534 23434935 10.2337/db12-0534 PMC3609593

[11] Cherney DZ, Reich HN, Scholey JW, Lai V, Slorach C, Zinman B, et al Systemic hemodynamic function in humans with type 1 diabetes treated with protein kinase Cbeta inhibition and renin-angiotensin system blockade: a pilot study. Can J Physiol Pharmacol. 2012;90(1):113–21. doi: 10.1139/y11-106 22188532 10.1139/y11-106

[12] Beckman JA, Goldfine AB, Gordon MB, Garrett LA, Creager MA. Inhibition of protein kinase Cbeta prevents impaired endothelium-dependent vasodilation caused by hyperglycemia in humans. Circ Res. 2002;90(1):107–11. 11786526 10.1161/hh0102.102359

[13] Schindler TH, Cardenas J, Prior JO, Facta AD, Kreissl MC, Zhang XL, et al Relationship between increasing body weight, insulin resistance, inflammation, adipocytokine leptin, and coronary circulatory function. J Am Coll Cardiol. 2006;47(6):1188–95. doi: 10.1016/j.jacc.2005.10.062 16545651 10.1016/j.jacc.2005.10.062

[14] Oflaz H, Ozbey N, Mantar F, Genchellac H, Mercanoglu F, Sencer E, et al Determination of endothelial function and early atherosclerotic changes in healthy obese women. Diabetes Nutr Metab. 2003;16(3):176–81. 14635735

[15] Ferri C, Desideri G, Valenti M, Bellini C, Pasin M, Santucci A, et al Early upregulation of endothelial adhesion molecules in obese hypertensive men. Hypertension. 1999;34(4 Pt 1):568–73. 10523328 10.1161/01.hyp.34.4.568

[16] Suh HS, Park YW, Kang JH, Lee SH, Lee HS, Shim KW. Vascular endothelial dysfunction tested by blunted response to endothelium-dependent vasodilation by salbutamol and its related factors in uncomplicated pre-menopausal obese women. Int J Obes. 2005;29(2):217–22. 10.1038/sj.ijo.0802642 15570314

[17] Joo Turoni C, Maranon RO, Felipe V, Bruno ME, Negrete A, Salas N, et al Arterial stiffness and endothelial function in obese children and adolescents and its relationship with cardiovascular risk factors. Horm Res Paediatr. 2013;80(4):281–6. doi: 10.1159/000354991 24060766 10.1159/000354991

[18] Hedvall Kallerman P, Hagman E, Edstedt Bonamy AK, Zemack H, Marcus C, Norman M, et al Obese children without comorbidities have impaired microvascular endothelial function. Acta Paediatr. 2014;103(4):411–7. doi: 10.1111/apa.12549 24372596 10.1111/apa.12549

[19] Pires A, Martins P, Paiva A, Pereira AM, Marques M, Castela E, et al Circulating endothelial progenitor cells in obese children and adolescents. J Pediatr (Rio J). 2015;91(6):560–6. 26321689 10.1016/j.jped.2015.01.011

[20] Bagi Z, Feher A, Cassuto J. Microvascular responsiveness in obesity: implications for therapeutic intervention. Br J Pharmacol. 2012;165(3):544–60. doi: 10.1111/j.1476-5381.2011.01606.x 21797844 10.1111/j.1476-5381.2011.01606.x PMC3315030

[21] Murray CD. The Physiological Principle of Minimum Work: I. The Vascular System and the Cost of Blood Volume. Proc Natl Acad Sci U S A. 1926;12(3):207–14. 16576980 10.1073/pnas.12.3.207 PMC1084489

[22] Crystal HA, Holman S, Lui YW, Baird AE, Yu H, Klein R, et al Association of the Fractal Dimension of Retinal Arteries and Veins with Quantitative Brain MRI Measures in HIV-Infected and Uninfected Women. PLoS One. 2016;11(5):e0154858 doi: 10.1371/journal.pone.0154858 27158911 10.1371/journal.pone.0154858 PMC4861324

[23] Gong W, Hu Y, Niu Y, Wang D, Wang Y, Li Y, et al Effects of longitudinal body mass index variability on microvasculature over 5 years in adult Chinese. Obesity (Silver Spring). 2016;24(3):743–9. 26833805 10.1002/oby.21398

[24] Gopinath B, Baur LA, Teber E, Liew G, Wong TY, Mitchell P. Effect of obesity on retinal vascular structure in pre-adolescent children. Int J Pediatr Obes. 2011;6(2–2):e353–9. doi: 10.3109/17477166.2010.500390 20883126 10.3109/17477166.2010.500390

[25] Taylor B, Rochtchina E, Wang JJ, Wong TY, Heikal S, Saw SM, et al Body mass index and its effects on retinal vessel diameter in 6-year-old children. Int J Obes. 2007;31(10):1527–33. 10.1038/sj.ijo.0803674 17607323

[26] Cheung CY, Zheng Y, Hsu W, Lee ML, Lau QP, Mitchell P, et al Retinal vascular tortuosity, blood pressure, and cardiovascular risk factors. Ophthalmology. 2011;118(5):812–8. doi: 10.1016/j.ophtha.2010.08.045 21146228 10.1016/j.ophtha.2010.08.045

[27] Grauslund J, Green A, Kawasaki R, Hodgson L, Sjolie AK, Wong TY. Retinal vascular fractals and microvascular and macrovascular complications in type 1 diabetes. Ophthalmology. 2010;117(7):1400–5. doi: 10.1016/j.ophtha.2009.10.047 20176399 10.1016/j.ophtha.2009.10.047

[28] Cheung N, Liew G, Lindley RI, Liu EY, Wang JJ, Hand P, et al Retinal fractals and acute lacunar stroke. Ann Neurol. 2010;68(1):107–11. doi: 10.1002/ana.22011 20582985 10.1002/ana.22011

[29] Sasongko MB, Wong TY, Nguyen TT, Cheung CY, Shaw JE, Kawasaki R, et al Retinal Vessel Tortuosity and Its Relation to Traditional and Novel Vascular Risk Markers in Persons with Diabetes. Curr Eye Res. 2016;41(4):551–7. doi: 10.3109/02713683.2015.1034371 26086266 10.3109/02713683.2015.1034371

[30] Knudtson MD, Lee KE, Hubbard LD, Wong TY, Klein R, Klein BE. Revised formulas for summarizing retinal vessel diameters. Curr Eye Res. 2003;27(3):143–9. 14562179 10.1076/ceyr.27.3.143.16049

[31] Bengtsson B, Krakau CET. Correction of optic disc measurements on fundus photographs. Graefes Arch Clin Exp Ophthalmol. 1992;230(1):24–8. 1547963 10.1007/BF00166758

[32] Masters BR. Fractal analysis of the vascular tree in the human retina. Annu Rev Biomed Eng. 2004;6:427–52. doi: 10.1146/annurev.bioeng.6.040803.140100 15255776 10.1146/annurev.bioeng.6.040803.140100

[33] Zamir M, Medeiros JA, Cunningham TK. Arterial bifurcations in the human retina. J Gen Physiol. 1979;74(4):537–48. 512630 10.1085/jgp.74.4.537 PMC2228563

[34] Ng E.Y.K., Rajendra Acharya U., Aurelio Campilho, Jasjit S. Suri. Image Analysis and Modelling in Ophthalmology. Boca Raton, Florida: CRC Press, Taylor & Francis Group; 2014.

[35] Hart WE, Goldbaum M, Cote B, Kube P, Nelson MR. Measurement and classification of retinal vascular tortuosity. Int J Med Inform. 1999;53(2–3):239–52. 10193892 10.1016/s1386-5056(98)00163-4

[36] Tai EL, Li LJ, Wan-Hazabbah WH, Wong TY, Shatriah I. Effect of Axial Eye Length on Retinal Vessel Parameters in 6 to 12-Year-Old Malay Girls. PLoS ONE. 2017;12(1):e0170014 doi: 10.1371/journal.pone.0170014 28107389 10.1371/journal.pone.0170014 PMC5249240

[37] Liang Y, Hou D, Zhao X, Wang L, Hu Y, Liu J, et al Childhood obesity affects adult metabolic syndrome and diabetes. Endocrine. 2015;50(1):87–92. doi: 10.1007/s12020-015-0560-7 25754912 10.1007/s12020-015-0560-7

[38] Rasmussen ML, Broe R, Frydkjaer-Olsen U, Olsen BS, Mortensen HB, Peto T, et al Retinal vascular geometry and its association to microvascular complications in patients with type 1 diabetes: the Danish Cohort of Pediatric Diabetes 1987 (DCPD1987). Graefes Arch Clin Exp Ophthalmol. 2017;255(2):293–9. doi: 10.1007/s00417-016-3454-3 27520462 10.1007/s00417-016-3454-3

[39] Gishti O, Jaddoe VW, Hofman A, Wong TY, Ikram MK, Gaillard R. Body fat distribution, metabolic and inflammatory markers and retinal microvasculature in school-age children. The Generation R Study. Int J Obes. 2015;39(10):1482–7. 10.1038/ijo.2015.99 26028060

[40] Xiao W, Gong W, Chen Q, Ding X, Chang B, He M. Association between body composition and retinal vascular caliber in children and adolescents. Invest Ophthalmol Vis Sci. 2015;56(2):705–10. doi: 10.1167/iovs.14-14946 25574054 10.1167/iovs.14-14946

[41] Mitchell P, Cheung N, de Haseth K, Taylor B, Rochtchina E, Islam FM, et al Blood pressure and retinal arteriolar narrowing in children. Hypertension. 2007;49(5):1156–62. doi: 10.1161/HYPERTENSIONAHA.106.085910 17372033 10.1161/HYPERTENSIONAHA.106.085910

[42] Gopinath B, Baur LA, Garnett S, Pfund N, Burlutsky G, Mitchell P. Body mass index and waist circumference are associated with blood pressure in preschool-aged children. Ann Epidemiol. 2011;21(5):351–7. doi: 10.1016/j.annepidem.2011.02.002 21458728 10.1016/j.annepidem.2011.02.002

[43] Frisbee JC, Delp MD. Vascular function in the metabolic syndrome and the effects on skeletal muscle perfusion: lessons from the obese Zucker rat. Essays Biochem. 2006;42:145–61. doi: 10.1042/bse0420145 17144886 10.1042/bse0420145

[44] Cawley T, Breslin E, Geraghty J, Osborne H, Docherty JR. Investigations of the function of the vascular endothelium in portal hypertensive rats. Pharmacology. 1995;51(6):381–90. 8966195 10.1159/000139350

[45] Brooks SD, DeVallance E, d'Audiffret AC, Frisbee SJ, Tabone LE, Shrader CD, et al Metabolic syndrome impairs reactivity and wall mechanics of cerebral resistance arteries in obese Zucker rats. Am J Physiol Heart Circ Physiol. 2015;309(11):H1846–59. doi: 10.1152/ajpheart.00691.2015 26475592 10.1152/ajpheart.00691.2015 PMC4698385

[46] Justo ML, Claro C, Vila E, Herrera MD, Rodriguez-Rodriguez R. Microvascular disorders in obese Zucker rats are restored by a rice bran diet. Nutr Metab Cardiovas. 2014;24(5):524–31. 10.1016/j.numecd.2013.10.032 24361072

[47] Gopinath B, Flood VM, Wang JJ, Smith W, Rochtchina E, Louie JC, et al Carbohydrate nutrition is associated with changes in the retinal vascular structure and branching pattern in children. Am J Clin Nutr. 2012;95(5):1215–22. doi: 10.3945/ajcn.111.031641 22456656 10.3945/ajcn.111.031641

[48] Kurniawan ED, Cheung N, Cheung CY, Tay WT, Saw SM, Wong TY. Elevated blood pressure is associated with rarefaction of the retinal vasculature in children. Invest Ophthalmol Vis Sci. 2012;53(1):470–4. doi: 10.1167/iovs.11-8835 22205607 10.1167/iovs.11-8835

[49] Yau JW, Kawasaki R, Islam FM, Shaw J, Zimmet P, Wang JJ, et al Retinal fractal dimension is increased in persons with diabetes but not impaired glucose metabolism: the Australian Diabetes, Obesity and Lifestyle (AusDiab) study. Diabetologia. 2010;53(9):2042–5. doi: 10.1007/s00125-010-1811-z 20523965 10.1007/s00125-010-1811-z

[50] Nagaoka T, Yoshida A. Noninvasive evaluation of wall shear stress on retinal microcirculation in humans. Invest Ophthalmol Vis Sci. 2006;47(3):1113–9. doi: 10.1167/iovs.05-0218 16505049 10.1167/iovs.05-0218

[51] Hamada M, Ogura Y, Miyamoto K, Nishiwaki H, Hiroshiba N, Honda Y. Retinal leukocyte behavior in experimental autoimmune uveoretinitis of rats. Exp Eye Res. 1997;65(3):445–50. doi: 10.1006/exer.1997.0361 9299181 10.1006/exer.1997.0361

[52] Auten RL, Davis JM. Oxygen toxicity and reactive oxygen species: the devil is in the details. Pediatr Res. 2009;66(2):121–7. doi: 10.1203/PDR.0b013e3181a9eafb 19390491 10.1203/PDR.0b013e3181a9eafb

[53] Bruyndonckx L, Hoymans VY, Lemmens K, Ramet J, Vrints CJ. Childhood obesity-related endothelial dysfunction: an update on pathophysiological mechanisms and diagnostic advancements. Pediatr Res. 2016;79(6):831–7. doi: 10.1038/pr.2016.22 26866906 10.1038/pr.2016.22

[54] Gimbrone MA Jr., Topper JN, Nagel T, Anderson KR, Garcia-Cardena G. Endothelial dysfunction, hemodynamic forces, and atherogenesis. Ann N Y Acad Sci. 2000;902:230–9; discussion 9–40. 10865843 10.1111/j.1749-6632.2000.tb06318.x

[55] Patton N, Pattie A, MacGillivray T, Aslam T, Dhillon B, Gow A, et al The association between retinal vascular network geometry and cognitive ability in an elderly population. Invest Ophthalmol Vis Sci. 2007;48(5):1995–2000. doi: 10.1167/iovs.06-1123 17460252 10.1167/iovs.06-1123

[56] Gameiro F, Perea MV, Ladera V, Rosa B, Garcia R. Executive functioning in obese individuals waiting for clinical treatment. Psicothema. 2017;29(1):61–6. doi: 10.7334/psicothema2016.202 28126060 10.7334/psicothema2016.202

[57] Witt N, Wong TY, Hughes AD, Chaturvedi N, Klein BE, Evans R, et al Abnormalities of retinal microvascular structure and risk of mortality from ischemic heart disease and stroke. Hypertension. 2006;47(5):975–81. doi: 10.1161/01.HYP.0000216717.72048.6c 16585415 10.1161/01.HYP.0000216717.72048.6c

[58] Benitez-Aguirre P, Craig ME, Sasongko MB, Jenkins AJ, Wong TY, Wang JJ, et al Retinal vascular geometry predicts incident retinopathy in young people with type 1 diabetes: a prospective cohort study from adolescence. Diabetes Care. 2011;34(7):1622–7. doi: 10.2337/dc10-2419 21593293 10.2337/dc10-2419 PMC3120178

[59] Naidu VV, Ismail K, Amiel S, Kohli R, Crosby-Nwaobi R, Sivaprasad S, et al Associations between Retinal Markers of Microvascular Disease and Cognitive Impairment in Newly Diagnosed Type 2 Diabetes Mellitus: A Case Control Study. PLoS ONE. 2016;11(1):e0147160 doi: 10.1371/journal.pone.0147160 26771382 10.1371/journal.pone.0147160 PMC4714814

[60] Kylstra JA, Wierzbicki T, Wolbarsht ML, Landers MB 3rd, Stefansson E. The relationship between retinal vessel tortuosity, diameter, and transmural pressure. Graefes Arch Clin Exp Ophthalmol. 1986;224(5):477–80. 3758696 10.1007/BF02173368

[61] Arciero J, Harris A, Siesky B, Amireskandari A, Gershuny V, Pickrell A, et al Theoretical analysis of vascular regulatory mechanisms contributing to retinal blood flow autoregulation. Invest Ophthalmol Vis Sci. 2013;54(8):5584–93. doi: 10.1167/iovs.12-11543 23847315 10.1167/iovs.12-11543 PMC3747715

[62] Chen HC, Patel V, Wiek J, Rassam SM, Kohner EM. Vessel diameter changes during the cardiac cycle. Eye (Lond). 1994;8 Pt 1):97–103. 8013728 10.1038/eye.1994.19

[63] Kawada T. Body mass index and fat mass by skin-fold thickness are good predictors for body fat composition change by dual-energy x-ray absorptiometry in obesity adolescent. Clin Nutr. 2016;35(4):983 doi: 10.1016/j.clnu.2016.04.023 27181527 10.1016/j.clnu.2016.04.023

