# Supplementary material for: Comparison of retinal vascular geometry in obese and non-obese children
Source: PLoS One. 2018 Feb 1;13(2):e0191434. doi: 10.1371/journal.pone.0191434 (PMC5794084; doi:10.1371/journal.pone.0191434)
Supplement: S1 Table — (DOCX) [file pone.0191434.s001.docx]

**S1 Table. Systemic, ocular and retinal vascular parameters of study subjects.**

| No | Age | Gender | Weight | Height | SBP | DBP | SE | AL | CRAE | CRVE | Dfa | Dfv | BCa | BCv | sTORTa | sTORTv | cTORTa | cTORTv |
| --- | --- | --- | --- | --- | --- | --- | --- | --- | --- | --- | --- | --- | --- | --- | --- | --- | --- | --- |
| 1 | 9 | F | 39.0 | 1.35 | 115 | 81 | -.38 | 22.32 | 156.196 | 240.311 | 1.097 | 1.204 | 0.999 | 2.11 | 1.087 | 1.110 | 6.0436 | 7.88466 |
| 2 | 12 | F | 52.0 | 1.52 | 106 | 67 | -.88 | 23.35 | 158.903 | 240.916 | 1.180 | 1.230 | 1.651 | 1.34 | 1.098 | 1.088 | 6.25376 | 5.29868 |
| 3 | 12 | F | 54.0 | 1.45 | 127 | 75 | -.88 | 23.34 | 163.681 | 249.683 | 1.268 | 1.188 | 1.282 | 1.31 | 1.085 | 1.097 | 5.43954 | 7.35125 |
| 4 | 9 | F | 19.0 | 1.06 | 111 | 64 | .00 | 22.55 | 185.126 | 227.591 | 1.152 | 1.113 | .971 | 1.25 | 1.085 | 1.100 | 6.40001 | 7.22663 |
| 5 | 12 | M | 33.0 | 1.21 | 111 | 64 | .00 | 23.50 | 168.883 | 229.519 | 1.208 | 1.112 | 1.461 | 1.59 | 1.097 | 1.103 | 5.72422 | 6.92593 |
| 6 | 12 | F | 59.0 | 1.54 | 118 | 81 | -.75 | 24.68 | 158.718 | 234.485 | 1.244 | 1.262 | 1.310 | 1.15 | 1.101 | 1.097 | 5.51371 | 4.96843 |
| 7 | 7 | M | 57.0 | 1.49 | 114 | 100 | .00 | 23.15 | 180.800 | 277.754 | 1.217 | 1.326 | 1.254 | 1.67 | 1.172 | 1.114 | 9.45557 | 7.01008 |
| 8 | 8 | M | 21.0 | 1.21 | 113 | 60 | .00 | 22.57 | 170.676 | 270.320 | 1.239 | 1.226 | 1.530 | 1.34 | 1.095 | 1.128 | 7.10844 | 8.88999 |
| 9 | 11 | F | 31.0 | 1.34 | 106 | 67 | .00 | 22.63 | 171.291 | 254.725 | 1.280 | 1.200 | 1.623 | 1.11 | 1.078 | 1.097 | 5.73217 | 6.16782 |
| 10 | 12 | M | 32.0 | 1.24 | 106 | 67 | .00 | 22.94 | 160.954 | 239.966 | 1.230 | 1.226 | 1.022 | 1.27 | 1.090 | 1.137 | 6.37836 | 7.65401 |
| 11 | 8 | F | 19.0 | 1.05 | 107 | 51 | .00 | 22.02 | 191.760 | 303.809 | 1.179 | 1.225 | 1.086 | 1.36 | 1.077 | 1.096 | 6.37836 | 6.71621 |
| 12 | 12 | F | 48.0 | 1.37 | 119 | 76 | -.50 | 21.68 | 174.222 | 282.901 | 1.267 | 1.257 | 1.663 | 1.37 | 1.093 | 1.103 | 7.07696 | 7.67593 |
| 13 | 11 | F | 50.0 | 1.53 | 100 | 57 | -.50 | 22.28 | 183.612 | 259.076 | 1.254 | 1.192 | 1.479 | 1.19 | 1.097 | 1.097 | 6.2646 | 6.71621 |
| 14 | 8 | F | 20.0 | 1.19 | 105 | 57 | -.25 | 21.28 | 168.590 | 247.524 | 1.248 | 1.219 | 1.284 | 1.13 | 1.127 | 1.109 | 7.83368 | 6.39268 |
| 15 | 12 | F | 47.0 | 1.46 | 105 | 52 | .13 | 21.37 | 201.113 | 244.190 | 1.300 | 1.262 | 1.443 | 1.01 | 1.097 | 1.083 | 7.18607 | 5.88516 |
| 16 | 10 | M | 23.0 | 1.26 | 82 | 52 | -.38 | 22.70 | 178.755 | 270.433 | 1.273 | 1.262 | 1.665 | 1.13 | 1.085 | 1.083 | 5.14798 | 6.08421 |
| 17 | 10 | M | 26.0 | 1.28 | 116 | 71 | -.50 | 23.83 | 159.741 | 224.715 | 1.215 | 1.157 | 1.431 | 1.29 | 1.090 | 1.088 | 6.59817 | 6.31074 |
| 18 | 10 | M | 34.5 | 1.31 | 120 | 60 | .00 | 23.05 | 154.821 | 251.222 | 1.141 | 1.229 | 2.312 | 1.37 | 1.111 | 1.091 | 7.57197 | 6.57261 |
| 19 | 11 | F | 31.0 | 1.41 | 107 | 67 | .00 | 23.29 | 152.587 | 231.644 | 1.217 | 1.230 | 1.468 | 1.25 | 1.086 | 1.109 | 5.04373 | 6.86706 |
| 20 | 6 | F | 20.5 | 1.22 | 106 | 64 | .00 | 23.52 | 155.538 | 252.143 | 1.216 | 1.206 | 1.378 | 1.17 | 1.087 | 1.095 | 5.34555 | 6.20111 |
| 21 | 12 | M | 36.5 | 1.50 | 112 | 54 | -.25 | 23.35 | 158.817 | 227.598 | 1.238 | 1.243 | 1.420 | 1.15 | 1.110 | 1.091 | 5.99384 | 5.32181 |
| 22 | 11 | F | 30.5 | 1.35 | 118 | 78 | -1.13 | 21.78 | 176.457 | 246.725 | 1.183 | 1.183 | 1.208 | 1.07 | 1.117 | 1.101 | 7.2241 | 6.73574 |
| 23 | 12 | F | 65.0 | 1.52 | 118 | 82 | -.13 | 23.49 | 154.978 | 253.128 | 1.247 | 1.203 | 1.267 | 1.12 | 1.088 | 1.081 | 5.9395 | 5.52648 |
| 24 | 12 | M | 77.0 | 1.47 | 126 | 73 | .00 | 24.12 | 165.558 | 230.088 | 1.161 | 1.110 | 1.013 | 1.34 | 1.080 | 1.093 | 6.14309 | 6.255 |
| 25 | 12 | F | 66.0 | 1.59 | 110 | 80 | .00 | 22.84 | 176.909 | 289.133 | 1.202 | 1.175 | 1.571 | 1.25 | 1.121 | 1.096 | 7.78035 | 7.53558 |
| 26 | 10 | M | 31.0 | 1.40 | 129 | 76 | .00 | 23.26 | 184.698 | 272.475 | 1.207 | 1.184 | 1.450 | 1.32 | 1.071 | 1.082 | 5.16415 | 6.7989 |
| 27 | 10 | F | 37.0 | 1.30 | 110 | 64 | .25 | 23.78 | 161.717 | 222.627 | 1.226 | 1.171 | 1.204 | 1.15 | 1.076 | 1.077 | 9.08926 | 8.05645 |
| 28 | 9 | F | 63.0 | 1.41 | 118 | 85 | -3.38 | 23.21 | 157.579 | 255.999 | 1.241 | 1.205 | 1.346 | 1.23 | 1.108 | 1.093 | 7.16975 | 7.32199 |
| 29 | 7 | M | 23.8 | 1.31 | 94 | 57 | -.25 | 22.53 | 173.707 | 258.391 | 1.236 | 1.216 | 1.108 | 1.25 | 1.103 | 1.106 | 7.12001 | 6.48215 |
| 30 | 10 | M | 28.0 | 1.45 | 69 | 55 | .00 | 24.14 | 174.144 | 233.030 | 1.250 | 1.173 | 1.578 | 1.29 | 1.091 | 1.086 | 5.62597 | 6.43887 |
| 31 | 12 | M | 44.7 | 1.40 | 119 | 75 | -1.50 | 23.69 | 166.217 | 253.283 | 1.174 | 1.188 | 1.288 | 1.01 | 1.078 | 1.098 | 4.96315 | 6.95392 |
| 32 | 9 | F | 19.0 | 1.16 | 95 | 43 | -.13 | 22.02 | 178.617 | 250.258 | 1.295 | 1.197 | 1.384 | 1.35 | 1.091 | 1.082 | 6.98838 | 5.71287 |
| 33 | 8 | F | 22.5 | 1.17 | 121 | 65 | .25 | 22.20 | 168.108 | 231.468 | 1.242 | 1.225 | 1.409 | 1.32 | 1.122 | 1.095 | 7.48759 | 5.92088 |
| 34 | 11 | M | 35.5 | 1.32 | 114 | 64 | .75 | 22.39 | 191.755 | 270.387 | 1.228 | 1.226 | 1.427 | 1.13 | 1.093 | 1.099 | 5.91826 | 6.62088 |
| 35 | 6 | M | 25.5 | 1.18 | 92 | 68 | .13 | 23.97 | 168.437 | 267.054 | 1.328 | 1.270 | 1.338 | 1.23 | 1.098 | 1.104 | 6.27032 | 6.13433 |
| 36 | 10 | F | 29.0 | 1.32 | 112 | 78 | .00 | 24.29 | 154.701 | 224.495 | 1.257 | 1.199 | 1.752 | 1.31 | 1.083 | 1.097 | 4.85467 | 5.98215 |
| 37 | 10 | M | 30.0 | 1.40 | 104 | 65 | .50 | 23.59 | 174.837 | 258.799 | 1.214 | 1.237 | 1.419 | 1.42 | 1.078 | 1.115 | 5.85955 | 7.14259 |
| 38 | 6 | M | 18.0 | 1.10 | 93 | 56 | .50 | 22.53 | 165.854 | 223.537 | 1.293 | 1.182 | 1.420 | 1.26 | 1.091 | 1.084 | 4.74827 | 5.67573 |
| 39 | 10 | M | 29.0 | 1.33 | 124 | 72 | .00 | 22.84 | 143.624 | 221.170 | 1.271 | 1.180 | 1.374 | 1.46 | 1.080 | 1.085 | 4.03751 | 5.83681 |
| 40 | 9 | M | 28.0 | 1.26 | 111 | 50 | .25 | 22.92 | 176.363 | 246.803 | 1.104 | 1.142 | 2.013 | 1.34 | 1.082 | 1.082 | 4.60595 | 6.60632 |
| 41 | 7 | F | 19.0 | 1.10 | 97 | 62 | .88 | 22.37 | 167.463 | 241.479 | 1.202 | 1.180 | 1.161 | 1.77 | 1.094 | 1.102 | 6.56738 | 6.77245 |
| 42 | 9 | F | 20.0 | 1.20 | 110 | 62 | -2.00 | 22.24 | 177.451 | 250.823 | 1.189 | 1.181 | 1.740 | 1.02 | 1.073 | 1.120 | 4.87369 | 6.5738 |
| 43 | 9 | M | 24.0 | 1.26 | 102 | 56 | -.25 | 23.53 | 169.670 | 240.920 | 1.190 | 1.262 | 1.912 | 1.39 | 1.079 | 1.110 | 5.12525 | 7.41843 |
| 44 | 7 | F | 22.0 | 1.16 | 98 | 64 | .13 | 22.70 | 171.353 | 246.440 | 1.159 | 1.098 | 1.795 | 1.02 | 1.070 | 1.083 | 4.30386 | 5.33481 |
| 45 | 7 | M | 21.0 | 1.20 | 119 | 78 | -.63 | 23.94 | 163.567 | 247.426 | 1.170 | 1.131 | 0.898 | 1.08 | 1.078 | 1.104 | 4.66936 | 5.88976 |
| 46 | 10 | F | 22.0 | 1.37 | 97 | 65 | .25 | 22.78 | 181.086 | 252.477 | 1.180 | 1.183 | 1.087 | 1.19 | 1.106 | 1.101 | 7.52379 | 6.22821 |
| 47 | 11 | F | 33.0 | 1.26 | 107 | 72 | -.88 | 23.69 | 159.875 | 261.978 | 1.216 | 1.195 | 1.524 | 1.31 | 1.093 | 1.096 | 6.35299 | 5.81558 |
| 48 | 11 | F | 31.0 | 1.38 | 115 | 56 | -2.00 | 23.23 | 186.282 | 249.836 | 1.198 | 1.127 | 1.315 | 1.37 | 1.092 | 1.103 | 6.09632 | 6.27644 |
| 49 | 8 | F | 18.0 | 1.19 | 117 | 77 | -1.13 | 23.25 | 154.586 | 223.993 | 1.171 | 1.221 | 1.280 | 1.30 | 1.097 | 1.076 | 6.0182 | 5.90842 |
| 50 | 7 | M | 29.0 | 1.24 | 108 | 72 | -.50 | 23.76 | 161.553 | 205.796 | 1.203 | 1.106 | 1.354 | 1.37 | 1.094 | 1.090 | 6.33747 | 5.3396 |
| 51 | 9 | F | 34.0 | 1.36 | 102 | 72 | -2.38 | 23.49 | 189.023 | 258.733 | 1.191 | 1.176 | 1.910 | 1.37 | 1.130 | 1.105 | 8.8487 | 6.8629 |
| 52 | 7 | M | 44.0 | 1.26 | 109 | 73 | -1.00 | 22.61 | 179.879 | 292.949 | 1.150 | 1.232 | 1.298 | 1.55 | 1.094 | 1.108 | 5.93598 | 7.27932 |
| 53 | 9 | F | 53.0 | 1.58 | 115 | 70 | -1.75 | 23.53 | 163.081 | 238.239 | 1.078 | 1.175 | 1.176 | 1.06 | 1.066 | 1.095 | 4.47747 | 6.51871 |
| 54 | 8 | F | 27.0 | 1.20 | 125 | 68 | -.88 | 23.10 | 139.363 | 247.836 | 1.093 | 1.110 | 2.397 | 1.18 | 1.079 | 1.082 | 4.85833 | 5.68649 |
| 55 | 8 | F | 33.0 | 1.25 | 91 | 63 | .13 | 22.92 | 189.174 | 285.617 | 1.235 | 1.248 | 1.482 | 1.22 | 1.072 | 1.110 | 4.89016 | 6.3473 |
| 56 | 8 | F | 30.0 | 1.34 | 98 | 72 | -1.50 | 24.12 | 183.678 | 272.948 | 1.100 | 1.107 | 1.328 | 1.52 | 1.083 | 1.073 | 4.50534 | 5.07023 |
| 57 | 8 | F | 58.0 | 1.37 | 121 | 78 | -.38 | 21.56 | 192.287 | 281.923 | 1.251 | 1.247 | 1.364 | 1.15 | 1.093 | 1.098 | 5.04549 | 7.28777 |
| 58 | 10 | F | 49.0 | 1.43 | 125 | 68 | -.13 | 23.11 | 172.171 | 264.526 | 1.271 | 1.237 | 1.850 | 1.21 | 1.134 | 1.101 | 9.50333 | 7.48278 |
| 59 | 8 | F | 46.0 | 1.38 | 123 | 73 | -.88 | 23.33 | 167.506 | 256.361 | 1.234 | 1.164 | 1.481 | 1.33 | 1.080 | 1.094 | 5.41092 | 6.07991 |
| 60 | 8 | F | 36.0 | 1.31 | 106 | 70 | .00 | 23.06 | 155.590 | 219.406 | 1.266 | 1.230 | 1.582 | 1.21 | 1.095 | 1.098 | 5.97512 | 5.25181 |
| 61 | 7 | F | 30.0 | 1.22 | 80 | 62 | .00 | 21.78 | 180.803 | 254.509 | 1.173 | 1.267 | 1.624 | 1.21 | 1.128 | 1.095 | 6.99426 | 7.06197 |
| 62 | 7 | F | 28.0 | 1.22 | 98 | 62 | .25 | 23.35 | 196.111 | 246.515 | 1.255 | 1.105 | 1.073 | 1.21 | 1.094 | 1.093 | 6.45189 | 6.31486 |
| 63 | 7 | F | 30.0 | 1.24 | 119 | 82 | -.88 | 23.54 | 183.539 | 242.897 | 1.189 | 1.200 | 1.141 | 1.17 | 1.084 | 1.075 | 6.19651 | 6.31715 |
| 64 | 11 | F | 41.0 | 1.41 | 111 | 71 | -1.13 | 22.54 | 172.144 | 257.712 | 1.237 | 1.187 | 1.323 | 1.21 | 1.097 | 1.126 | 6.14383 | 6.41672 |
| 65 | 8 | M | 43.0 | 1.31 | 120 | 98 | .13 | 23.13 | 162.284 | 228.577 | 1.257 | 1.146 | 1.562 | 1.43 | 1.114 | 1.099 | 7.97671 | 6.73989 |
| 66 | 10 | M | 45.0 | 1.43 | 125 | 70 | -.25 | 23.67 | 167.706 | 266.607 | 1.244 | 1.214 | 1.326 | 1.32 | 1.082 | 1.076 | 5.49973 | 5.71527 |
| 67 | 9 | F | 35.0 | 1.33 | 96 | 56 | -1.25 | 22.27 | 178.657 | 255.002 | 1.201 | 1.194 | 1.388 | 1.08 | 1.094 | 1.084 | 5.40723 | 6.68028 |
| 68 | 9 | F | 42.0 | 1.38 | 121 | 86 | -1.00 | 23.75 | 165.060 | 234.911 | 1.055 | 1.078 | 2.660 | 2.97 | 1.125 | 1.072 | 6.47578 | 4.99375 |
| 69 | 9 | F | 42.0 | 1.31 | 116 | 71 | -1.00 | 22.78 | 151.411 | 261.446 | 1.188 | 1.179 | 1.727 | 1.21 | 1.100 | 1.090 | 7.23139 | 6.57076 |
| 70 | 9 | F | 39.0 | 1.34 | 105 | 64 | .00 | 24.05 | 169.065 | 270.606 | 1.186 | 1.156 | 1.424 | 1.99 | 1.095 | 1.100 | 7.18547 | 8.16825 |
| 71 | 9 | F | 41.0 | 1.28 | 105 | 64 | -.38 | 23.84 | 146.640 | 250.319 | 1.179 | 1.172 | 1.106 | 1.21 | 1.077 | 1.086 | 5.51142 | 6.52357 |
| 72 | 10 | F | 69.0 | 1.42 | 122 | 69 | -.75 | 23.66 | 143.317 | 215.119 | 1.027 | 1.038 | 0.999 | 0.99 | 1.081 | 1.083 | 6.63784 | 8.6254 |
| 73 | 12 | F | 56.0 | 1.50 | 119 | 70 | -.63 | 22.38 | 164.177 | 238.409 | 1.181 | 1.296 | 1.300 | 1.29 | 1.111 | 1.095 | 7.22973 | 6.71487 |
| 74 | 11 | F | 58.0 | 1.55 | 102 | 58 | -2.38 | 23.94 | 162.299 | 235.011 | 1.170 | 1.207 | 2.019 | 1.28 | 1.083 | 1.086 | 5.25547 | 7.53754 |
| 75 | 11 | F | 49.0 | 1.48 | 95 | 62 | -.25 | 22.57 | 176.954 | 249.349 | 1.181 | 1.145 | 1.590 | 1.36 | 1.087 | 1.117 | 7.00725 | 7.84182 |
| 76 | 9 | F | 44.0 | 1.35 | 105 | 70 | -1.00 | 23.53 | 159.556 | 260.873 | 1.179 | 1.220 | 1.219 | .94 | 1.077 | 1.088 | 5.45731 | 5.30923 |
| 77 | 10 | F | 36.0 | 1.35 | 104 | 69 | .00 | 23.55 | 165.816 | 242.361 | 1.265 | 1.173 | 1.246 | 1.17 | 1.119 | 1.111 | 7.50061 | 7.12672 |
| 78 | 10 | F | 48.0 | 1.53 | 105 | 71 | .25 | 23.87 | 180.240 | 250.013 | 1.180 | 1.117 | 1.706 | 0.99 | 1.082 | 1.093 | 6.25923 | 6.14052 |
| 79 | 10 | F | 51.0 | 1.48 | 105 | 71 | -.25 | 22.52 | 161.789 | 256.878 | 1.178 | 1.152 | 1.399 | 1.40 | 1.087 | 1.132 | 6.39663 | 9.75453 |
| 80 | 10 | F | 42.0 | 1.38 | 126 | 70 | .63 | 23.85 | 162.344 | 251.177 | 1.229 | 1.234 | 1.706 | .94 | 1.088 | 1.086 | 6.41354 | 6.75973 |
| 81 | 10 | F | 55.0 | 1.46 | 116 | 84 | .50 | 22.50 | 164.452 | 257.735 | 1.195 | 1.189 | 1.889 | 1.17 | 1.109 | 1.111 | 5.62085 | 6.76552 |
| 82 | 10 | F | 43.0 | 1.46 | 109 | 61 | -.50 | 24.50 | 166.955 | 248.200 | 1.263 | 1.148 | 1.259 | 1.65 | 1.095 | 1.081 | 8.62225 | 6.4133 |
| 83 | 8 | F | 30.0 | 1.30 | 115 | 62 | -.50 | 23.16 | 180.371 | 265.636 | 1.299 | 1.152 | 1.384 | 1.09 | 1.148 | 1.114 | 9.32325 | 7.05161 |
| 84 | 11 | F | 57.0 | 1.41 | 130 | 75 | -.38 | 22.45 | 184.148 | 257.747 | 1.201 | 1.186 | 1.603 | 1.23 | 1.093 | 1.088 | 7.31159 | 5.41018 |
| 85 | 11 | F | 52.0 | 1.43 | 103 | 64 | -.38 | 23.44 | 169.632 | 244.864 | 1.190 | 1.157 | 1.001 | 1.16 | 1.108 | 1.103 | 6.95194 | 6.95526 |
| 86 | 11 | F | 51.0 | 1.48 | 128 | 63 | .25 | 24.33 | 161.306 | 243.023 | 1.190 | 1.214 | 1.351 | 1.11 | 1.101 | 1.088 | 6.00789 | 6.98485 |
| 87 | 11 | F | 52.0 | 1.44 | 114 | 56 | -.38 | 22.56 | 182.110 | 256.383 | 1.289 | 1.222 | 1.757 | 1.07 | 1.089 | 1.092 | 7.00689 | 5.58148 |
| 88 | 11 | F | 49.0 | 1.47 | 99 | 69 | -.50 | 21.89 | 195.848 | 260.975 | 1.240 | 1.170 | 1.576 | 1.06 | 1.151 | 1.082 | 7.73405 | 6.14412 |
| 89 | 11 | F | 49.0 | 1.52 | 128 | 69 | .00 | 22.84 | 187.135 | 264.771 | 1.188 | 1.149 | 1.499 | 1.34 | 1.087 | 1.088 | 6.97485 | 7.95265 |
| 90 | 8 | F | 46.0 | 1.25 | 126 | 73 | .00 | 22.89 | 147.975 | 257.609 | 1.153 | 1.192 | 0.999 | 1.30 | 1.092 | 1.120 | 6.78254 | 7.90476 |
| 91 | 11 | F | 48.0 | 1.41 | 103 | 64 | -.13 | 22.60 | 151.556 | 254.792 | 1.252 | 1.260 | 1.248 | 1.40 | 1.093 | 1.092 | 6.27963 | 5.72908 |
| 92 | 10 | F | 42.0 | 1.45 | 99 | 65 | .13 | 23.32 | 180.576 | 265.698 | 1.318 | 1.274 | 1.232 | 1.24 | 1.111 | 1.072 | 6.25964 | 4.96839 |
| 93 | 9 | F | 49.0 | 1.38 | 99 | 69 | -.50 | 22.81 | 160.332 | 245.500 | 1.172 | 1.210 | 1.499 | 1.02 | 1.188 | 1.093 | 9.60279 | 7.38668 |
| 94 | 12 | F | 48.0 | 1.48 | 94 | 64 | -.88 | 23.12 | 175.185 | 253.328 | 1.280 | 1.262 | 1.306 | 1.31 | 1.124 | 1.109 | 6.65321 | 6.55121 |
| 95 | 12 | F | 54.0 | 1.54 | 109 | 64 | .00 | 22.62 | 167.540 | 245.833 | 1.145 | 1.189 | 1.603 | 1.20 | 1.104 | 1.085 | 6.23042 | 5.35317 |
| 96 | 10 | M | 35.0 | 1.40 | 88 | 75 | -.13 | 23.24 | 162.827 | 238.803 | 1.213 | 1.219 | 1.786 | 1.32 | 1.070 | 1.080 | 4.97079 | 5.41012 |
| 97 | 6 | F | 16.0 | 1.04 | 91 | 63 | .50 | 21.72 | 185.294 | 277.276 | 1.239 | 1.227 | 1.319 | 1.34 | 1.098 | 1.102 | 5.7262 | 6.35435 |
| 98 | 8 | F | 21.0 | 1.13 | 100 | 64 | -.25 | 22.11 | 164.637 | 244.887 | 1.260 | 1.252 | 1.093 | 1.55 | 1.083 | 1.106 | 5.64353 | 6.50398 |
| 99 | 10 | M | 25.0 | 1.30 | 120 | 74 | .25 | 23.57 | 157.657 | 242.941 | 1.282 | 1.172 | 1.256 | 1.16 | 1.092 | 1.079 | 6.95623 | 6.16858 |
| 100 | 9 | M | 30.0 | 1.46 | 118 | 78 | -.13 | 22.66 | 170.674 | 229.623 | 1.267 | 1.169 | 1.327 | 1.24 | 1.095 | 1.100 | 5.90807 | 7.03566 |
| 101 | 12 | F | 54.0 | 1.54 | 113 | 77 | .00 | 22.62 | 169.369 | 242.680 | 1.162 | 1.192 | 1.463 | 1.21 | 1.107 | 1.084 | 6.22488 | 5.75664 |
| 102 | 7 | F | 27.0 | 1.23 | 100 | 52 | .50 | 22.39 | 152.420 | 234.465 | 1.187 | 1.174 | 1.225 | 1.25 | 1.140 | 1.119 | 7.95774 | 6.21615 |
| 103 | 7 | F | 29.0 | 1.23 | 88 | 55 | -1.13 | 22.95 | 181.756 | 261.520 | 1.163 | 1.068 | 0.904 | 0.99 | 1.092 | 1.084 | 6.10694 | 5.4146 |
| 104 | 7 | F | 35.0 | 1.20 | 85 | 65 | -.38 | 21.90 | 188.067 | 255.671 | 1.224 | 1.232 | 1.376 | 1.46 | 1.115 | 1.106 | 6.71432 | 6.62505 |
| 105 | 7 | F | 33.0 | 1.23 | 74 | 52 | .13 | 22.18 | 167.224 | 253.093 | 1.289 | 1.233 | 1.257 | 1.24 | 1.153 | 1.088 | 6.92225 | 6.66308 |
| 106 | 7 | F | 26.0 | 1.14 | 102 | 74 | -.88 | 22.01 | 155.324 | 239.411 | 1.249 | 1.198 | 1.602 | 1.22 | 1.101 | 1.088 | 5.59298 | 7.25161 |
| 107 | 7 | F | 29.0 | 1.24 | 110 | 67 | -.75 | 23.07 | 173.125 | 240.998 | 1.180 | 1.222 | 1.759 | 1.43 | 1.076 | 1.096 | 4.95565 | 6.50734 |
| 108 | 7 | F | 34.0 | 1.24 | 94 | 64 | -.13 | 22.42 | 175.523 | 272.546 | 1.217 | 1.272 | 1.336 | 1.21 | 1.143 | 1.093 | 8.4778 | 6.6516 |
| 109 | 8 | F | 30.0 | 1.15 | 100 | 62 | .25 | 22.16 | 152.676 | 222.143 | 1.206 | 1.144 | 1.152 | 1.14 | 1.094 | 1.099 | 6.17405 | 6.21058 |
| 110 | 10 | F | 45.5 | 1.24 | 113 | 77 | -.75 | 23.45 | 169.758 | 270.244 | 1.252 | 1.178 | 1.836 | 1.16 | 1.126 | 1.085 | 6.30326 | 4.97744 |
| 111 | 7 | F | 27.0 | 1.22 | 116 | 81 | -1.00 | 22.86 | 158.752 | 227.773 | 1.168 | 1.156 | 1.678 | 1.07 | 1.107 | 1.081 | 7.99239 | 7.13483 |
| 112 | 12 | F | 56.0 | 1.50 | 113 | 77 | -.63 | 22.38 | 166.747 | 233.221 | 1.168 | 1.234 | 1.177 | 1.39 | 1.097 | 1.090 | 5.99746 | 6.78746 |
| 113 | 12 | F | 48.0 | 1.48 | 94 | 64 | -.88 | 23.12 | 175.558 | 254.766 | 1.267 | 1.269 | 1.268 | 1.53 | 1.118 | 1.112 | 6.70658 | 6.64267 |
| 114 | 9 | F | 39.0 | 1.34 | 95 | 55 | -1.00 | 24.00 | 181.067 | 250.214 | 1.160 | 1.186 | 1.738 | 1.22 | 1.129 | 1.090 | 7.2549 | 5.62908 |
| 115 | 8 | M | 29.0 | 1.23 | 88 | 58 | .13 | 24.86 | 163.876 | 222.283 | 1.243 | 1.222 | 1.377 | 1.48 | 1.098 | 1.099 | 6.44824 | 6.75899 |
| 116 | 7 | M | 29.0 | 1.22 | 88 | 58 | .00 | 23.60 | 165.772 | 241.291 | 1.203 | 1.292 | 1.099 | 2.03 | 1.086 | 1.085 | 5.81640 | 5.97162 |
| 117 | 7 | M | 26.0 | 1.13 | 97 | 50 | -1.00 | 23.49 | 146.995 | 244.225 | 1.195 | 1.203 | 1.246 | 1.44 | 1.099 | 1.108 | 6.60654 | 6.03119 |
| 118 | 7 | M | 33.0 | 1.19 | 103 | 62 | -.88 | 24.00 | 152.099 | 263.393 | 1.151 | 1.227 | 0.944 | 1.98 | 1.115 | 1.110 | 6.72999 | 9.23448 |
| 119 | 7 | M | 34.0 | 1.24 | 106 | 52 | -.13 | 23.02 | 174.995 | 242.274 | 1.194 | 1.198 | 1.566 | 1.36 | 1.101 | 1.113 | 5.96218 | 6.36087 |
| 120 | 7 | M | 30.0 | 1.20 | 79 | 53 | -2.88 | 24.50 | 166.908 | 252.325 | 1.295 | 1.220 | 1.254 | 1.23 | 1.081 | 1.082 | 5.60506 | 6.21032 |
| 121 | 9 | M | 42.0 | 1.39 | 106 | 60 | -1.00 | 22.78 | 159.945 | 258.942 | 1.235 | 1.221 | 1.318 | 1.17 | 1.088 | 1.137 | 5.24256 | 7.45533 |
| 122 | 9 | M | 38.0 | 1.30 | 115 | 77 | -.63 | 23.49 | 165.169 | 255.690 | 1.257 | 1.192 | 1.536 | 1.44 | 1.126 | 1.102 | 7.23318 | 6.87793 |
| 123 | 9 | M | 44.0 | 1.28 | 116 | 75 | -.63 | 23.59 | 174.519 | 263.161 | 1.175 | 1.209 | 1.580 | 1.55 | 1.095 | 1.113 | 6.29002 | 6.44443 |
| 124 | 9 | M | 51.0 | 1.37 | 113 | 77 | -.25 | 23.81 | 157.161 | 229.295 | 1.164 | 1.206 | 1.142 | 1.10 | 1.093 | 1.103 | 7.38276 | 6.39315 |
| 125 | 9 | M | 39.0 | 1.26 | 116 | 67 | .50 | 21.97 | 169.019 | 236.986 | 1.209 | 1.124 | 1.589 | 1.29 | 1.083 | 1.086 | 6.46566 | 7.01503 |
| 126 | 9 | M | 37.0 | 1.32 | 88 | 45 | -.25 | 22.06 | 160.562 | 237.095 | 1.231 | 1.267 | 1.537 | 1.27 | 1.098 | 1.091 | 5.96228 | 6.22617 |
| 127 | 9 | M | 35.0 | 1.32 | 104 | 68 | -3.25 | 24.46 | 146.232 | 224.161 | 1.238 | 1.300 | 1.330 | 1.40 | 1.102 | 1.096 | 7.17732 | 7.07526 |
| 128 | 11 | M | 51.0 | 1.45 | 113 | 70 | -1.00 | 24.25 | 150.348 | 272.943 | 1.238 | 1.241 | 2.070 | 1.09 | 1.099 | 1.122 | 7.04777 | 6.96641 |
| 129 | 10 | M | 43.0 | 1.37 | 123 | 77 | -.50 | 23.33 | 162.901 | 239.951 | 1.163 | 1.168 | 1.455 | 1.34 | 1.112 | 1.101 | 7.89805 | 7.46497 |
| 130 | 11 | M | 48.0 | 1.50 | 102 | 67 | -1.25 | 22.01 | 193.533 | 254.751 | 1.215 | 1.246 | 1.335 | 1.14 | 1.099 | 1.094 | 5.86438 | 5.61907 |
| 131 | 9 | M | 50.0 | 1.20 | 110 | 60 | -.63 | 23.32 | 152.054 | 256.115 | 1.188 | 1.164 | 1.446 | 1.42 | 1.083 | 1.110 | 6.13791 | 7.30695 |
| 132 | 11 | M | 66.0 | 1.43 | 122 | 82 | .00 | 24.11 | 170.319 | 252.716 | 1.173 | 1.246 | 1.463 | 1.34 | 1.080 | 1.100 | 6.40104 | 6.75012 |
| 133 | 11 | M | 42.0 | 1.39 | 119 | 58 | -.25 | 24.10 | 158.913 | 254.164 | 1.219 | 1.213 | 1.219 | 1.13 | 1.066 | 1.087 | 5.56182 | 6.63558 |
| 134 | 11 | M | 36.0 | 1.29 | 116 | 64 | .63 | 21.94 | 173.322 | 246.528 | 1.211 | 1.214 | 1.391 | 1.63 | 1.093 | 1.097 | 6.72059 | 6.33414 |
| 135 | 11 | M | 45.0 | 1.44 | 107 | 72 | .38 | 23.77 | 167.561 | 246.991 | 1.252 | 1.146 | 1.433 | 1.41 | 1.089 | 1.102 | 6.34911 | 7.0496 |
| 136 | 11 | M | 49.0 | 1.37 | 119 | 67 | -1.13 | 24.61 | 143.921 | 230.251 | 1.242 | 1.200 | 1.542 | 1.62 | 1.100 | 1.087 | 6.58284 | 6.99957 |
| 137 | 11 | M | 54.0 | 1.35 | 111 | 64 | .00 | 25.33 | 151.732 | 227.172 | 1.159 | 1.152 | 1.289 | 1.16 | 1.109 | 1.091 | 6.40206 | 7.20509 |
| 138 | 11 | M | 49.0 | 1.48 | 93 | 61 | -4.13 | 25.08 | 150.734 | 252.792 | 1.206 | 1.223 | 1.465 | 1.32 | 1.068 | 1.092 | 4.92751 | 6.41569 |
| 139 | 10 | M | 39.0 | 1.38 | 106 | 70 | -.50 | 22.86 | 165.739 | 251.643 | 1.262 | 1.162 | 1.554 | 1.37 | 1.101 | 1.078 | 5.44074 | 5.52319 |
| 140 | 11 | M | 58.0 | 1.49 | 108 | 67 | -.25 | 23.16 | 169.642 | 267.850 | 1.264 | 1.275 | 1.286 | 1.20 | 1.092 | 1.085 | 4.94057 | 6.95077 |
| 141 | 12 | M | 54.0 | 1.41 | 129 | 77 | -.25 | 23.06 | 188.141 | 268.881 | 1.226 | 1.239 | 1.470 | 1.31 | 1.105 | 1.078 | 7.74083 | 5.97616 |
| 142 | 12 | M | 51.0 | 1.51 | 120 | 64 | -.13 | 22.66 | 205.042 | 271.639 | 1.238 | 1.189 | 1.434 | 1.39 | 1.100 | 1.154 | 7.09654 | 6.87086 |
| 143 | 12 | M | 52.0 | 1.54 | 122 | 89 | .63 | 22.59 | 160.398 | 286.262 | 1.243 | 1.225 | 1.661 | 1.13 | 1.089 | 1.105 | 5.66564 | 6.63958 |
| 144 | 12 | M | 46.0 | 1.46 | 117 | 68 | .88 | 22.59 | 180.698 | 280.155 | 1.235 | 1.275 | 1.363 | 1.24 | 1.074 | 1.116 | 5.8455 | 6.68756 |
| 145 | 12 | M | 53.0 | 1.49 | 110 | 72 | -1.25 | 24.54 | 155.751 | 241.483 | 1.217 | 1.253 | 1.514 | 1.45 | 1.076 | 1.098 | 5.37016 | 6.77275 |
| 146 | 12 | M | 50.0 | 1.43 | 115 | 78 | -.38 | 23.42 | 157.254 | 243.458 | 1.214 | 1.214 | 1.204 | 1.45 | 1.094 | 1.098 | 6.15742 | 7.69771 |
| 147 | 10 | M | 41.0 | 1.28 | 96 | 61 | -1.63 | 22.79 | 161.714 | 249.561 | 1.193 | 1.265 | 1.447 | 1.18 | 1.102 | 1.110 | 5.91263 | 5.7923 |
| 148 | 11 | M | 56.0 | 1.41 | 113 | 77 | -.88 | 23.40 | 170.205 | 250.454 | 1.176 | 1.171 | 1.813 | 1.09 | 1.083 | 1.084 | 5.57268 | 5.51136 |
| 149 | 11 | M | 65.0 | 1.45 | 115 | 65 | -.25 | 24.03 | 143.242 | 222.245 | 1.220 | 1.168 | 1.568 | 1.88 | 1.114 | 1.101 | 6.98023 | 6.92549 |
| 150 | 11 | M | 59.0 | 1.43 | 114 | 71 | .00 | 24.05 | 162.949 | 255.658 | 1.243 | 1.218 | 1.374 | 1.10 | 1.104 | 1.090 | 6.94923 | 6.75094 |
| 151 | 10 | M | 50.0 | 1.36 | 91 | 59 | -1.00 | 22.84 | 157.790 | 251.975 | 1.151 | 1.168 | 2.859 | 1.25 | 1.068 | 1.092 | 4.85485 | 6.0092 |
| 152 | 10 | M | 42.0 | 1.38 | 124 | 86 | .25 | 23.49 | 169.277 | 275.041 | 1.177 | 1.252 | 1.246 | 1.63 | 1.095 | 1.089 | 5.94357 | 7.30766 |
| 153 | 10 | M | 86.0 | 1.46 | 103 | 60 | -.38 | 21.87 | 173.886 | 269.357 | 1.214 | 1.171 | 1.726 | 1.12 | 1.089 | 1.102 | 5.68076 | 7.00862 |
| 154 | 10 | M | 50.0 | 1.38 | 112 | 71 | .38 | 23.66 | 151.935 | 222.553 | 1.279 | 1.252 | 1.544 | 1.36 | 1.097 | 1.090 | 6.31422 | 6.31727 |
| 155 | 10 | M | 45.0 | 1.39 | 103 | 62 | -1.00 | 23.63 | 179.914 | 257.451 | 1.264 | 1.238 | 1.428 | 1.27 | 1.155 | 1.102 | 8.3695 | 7.13967 |
| 156 | 10 | M | 48.0 | 1.39 | 117 | 77 | .63 | 22.40 | 143.319 | 229.597 | 1.215 | 1.266 | 1.269 | 1.43 | 1.074 | 1.103 | 4.90703 | 7.38565 |
| 157 | 10 | M | 50.0 | 1.43 | 104 | 56 | -.38 | 23.69 | 156.865 | 261.981 | 1.165 | 1.244 | 1.457 | 1.09 | 1.100 | 1.094 | 5.98688 | 7.80689 |
| 158 | 10 | M | 55.0 | 1.44 | 112 | 71 | -.50 | 23.19 | 156.999 | 253.373 | 1.170 | 1.167 | 1.241 | 1.28 | 1.077 | 1.087 | 6.20193 | 6.19365 |
| 159 | 10 | M | 44.0 | 1.41 | 118 | 81 | .25 | 24.16 | 149.382 | 226.925 | 1.219 | 1.234 | 1.000 | 1.40 | 1.090 | 1.085 | 6.40326 | 6.56107 |
| 160 | 10 | M | 42.0 | 1.36 | 113 | 88 | .25 | 22.25 | 179.847 | 271.169 | 1.255 | 1.201 | 1.244 | 1.41 | 1.100 | 1.118 | 6.49959 | 7.86327 |
| 161 | 10 | M | 34.0 | 1.30 | 98 | 53 | -1.25 | 22.42 | 168.904 | 245.299 | 1.181 | 1.212 | 1.349 | 1.18 | 1.076 | 1.086 | 4.95067 | 5.97345 |
| 162 | 10 | M | 38.0 | 1.30 | 118 | 68 | .38 | 23.05 | 159.696 | 231.701 | 1.242 | 1.222 | 1.431 | 1.33 | 1.085 | 1.120 | 6.45857 | 8.08315 |
| 163 | 10 | M | 48.0 | 1.39 | 114 | 83 | -.13 | 24.11 | 153.687 | 227.085 | 1.190 | 1.230 | 1.340 | 1.40 | 1.082 | 1.085 | 5.65845 | 6.03714 |
| 164 | 10 | M | 62.0 | 1.49 | 112 | 61 | -.75 | 23.61 | 176.421 | 253.421 | 1.217 | 1.208 | 1.623 | 1.35 | 1.092 | 1.099 | 7.29259 | 6.82701 |
| 165 | 10 | M | 51.0 | 1.35 | 101 | 61 | .13 | 22.99 | 172.306 | 274.686 | 1.235 | 1.246 | 1.640 | 1.20 | 1.071 | 1.088 | 4.67117 | 5.48752 |
| 166 | 8 | M | 47.0 | 1.35 | 117 | 75 | -.13 | 22.53 | 189.415 | 287.290 | 1.209 | 1.216 | 1.485 | 1.42 | 1.083 | 1.085 | 5.86059 | 6.56651 |

M, male; F, female; SBP, systolic blood pressure; DBP, diastolic blood pressure; SE, spherical equivalent; AL, axial length; CRAE, central retinal arteriolar equivalent; CRVE, central retinal venular equivalent; DFa, arteriolar fractal dimension; Dfv, venular fractal dimension; BCa, arteriolar branching coefficient; BCv, venular branching coefficient; sTORTa, arteriolar simple tortuosity; sTORTv, venular simple tortuosity; cTORTa, arteriolar curvature tortuosity; cTORTV, venular curvature tortuosity
